# Mammalian Cells Change Volume during Mitosis

**DOI:** 10.1371/journal.pone.0001477

**Published:** 2008-01-23

**Authors:** Emmanuel Boucrot, Tomas Kirchhausen

**Affiliations:** Department of Cell Biology and Immune Disease Institute, Harvard Medical School, Boston, Massachusetts, United States of America; Ordway Research Institute, United States of America

## Abstract

Using single cell-imaging methods we have found that the volume of adherent cells grown in culture decreases as the cells rounds when it enters mitosis. A minimal volume is reached at metaphase. Rapid volume recovery initiates before abscission as cells make the transition from metaphase to cytokinesis. These volume changes are simultaneous with the rapid surface area decrease and recovery observed in mitotic cells [1].

## Introduction

Adherent or non-adherent eukaryotic cells dramatically decrease the constitutive recycling of membranes as they enter prophase, leading to a significant loss of surface area. The area reaches a minimum at metaphase [1]. Because endocytosis remains constant, there is a loss of surface area that can be up to 6–8 fold. The internalized membrane accumulates in endosomal structures [1]. If we assume that the endocytic carriers have an average diameter of 100 nm, then their entrapped volume would increase the total cell volume by ∼3–5 %. As cells continue through anaphase, telophase and cytokinesis, membrane recycling starts again, resulting in rapid and complete recovery of surface area even before executing abscission [1]. We have adapted the single-cell imaging approach to monitor how the intracellular volume changes as a cell undergoes mitosis. Unexpectedly we found that (i) as cells approach metaphase their volume decreases and (ii) towards the end of cytokinesis but prior to abscission, the combined volume of the two daughter cells equals that of their mother cell during prophase. These volumes changes might influence the cytosolic protein concentration with the potential to influence enzymatic rates involved in regulating signaling checkpoint(s) during cell division.

## Results and Discussion

We determined changes of cellular volume during the cell cycle in BSC1 cells stably expressing EGFP-CAAX, a fluorescent chimeric protein of EGFP fused at its C-teminus to a CAAX prenylation motif. Prenylation targets this reporter protein to the cytosolic side of the plasma membrane [2]. Because the abundance of the free cytosolic form is relatively low, this fluorescent reporter sharply demarcates the location of the plasma membrane. We have previously used this property to follow changes of surface area during cell division [1]. As a simple extension of the same imaging approach, we determined the three dimensional distribution of EGFP-CAAX at the cell surface and then used this information to calculate the volume of a given cell. The enclosed volume is calculated by first defining on each plane a two-dimensional mask corresponding to the outline of the EGFP-CAAX fluorescence signal, followed by integration along the z-axis of all enclosed areas. Sequential imaging planes were rapidly acquired every 0.25 µm along the z-axis (100 ms exposure, total duration ∼5 s) using a spinning disk confocal head. The images were significantly deblured by using a computer-driven spherical aberration correction unit (SAC) optically coupled to a confocal head allowing us to attain the expected optical ∼0.8 µm resolution along the z-axis (manuscript in preparation).

**Figure 1:**
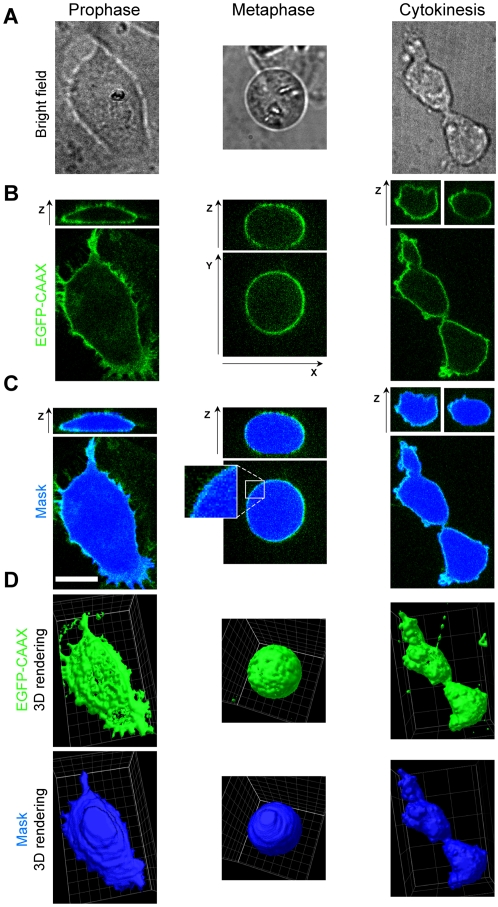
Single cell imaging during mitosis. (A) Representative images of the same BSC1 cell visualized by bright field phase contrast illumination during prophase, metaphase or cytokinesis (B) Ortogonal fluorescent views corresponding to the distribution of EGFP-CAAX in the same cell imaged using the confocal spinning head. The stacks along the z-axis correspond to sequential optical sections acquired 0.25 µm apart. The panels show the fluorescence signal along the z-axis (top) along a single confocal plane positioned approximately in the middle of the cell. Bar, 10 µm. (C) The mask (light blue) corresponds to the position of the EGFP-CAAX signal calculated for each confocal plane (see Methods). The outline of the outer boundary determined for any given plane defines the enclosed area for such plane (dark blue). (D) Three-dimensional rendition comparing the distribution of EGFP-CAAX (green) and the calculated cell volume (blue).

Cells entering prophase were readily identified by the appearance of their chromosomes observed using bright field phase contrast illumination. The spatial distribution of EGFP-CAAX of a selected cell was immediately determined by a first round of three-dimensional fluorescence imaging, followed by a second imaging series after the cell reached metaphase, and a final one during cytokinesis, ∼45 min after the onset of anaphase, at time at which telophase has ended but abscission has not yet occurred. Representative examples of images from a cell (out of 10 analyzed) at these three stages are shown in Figure 1 (bright field (A) and fluorescence (B)). We used the outline of the EGFP-CAAX signal (Figure 1B, green) defined by the most external pixels to generate a mask (Figure 1C, blue). Three-dimensional rendering of the data shows that the masking procedure faithfully follows the cell boundary (Figure 1D, green) used to define the enclosed volume (Figure 1D, blue).

**Figure 2:**
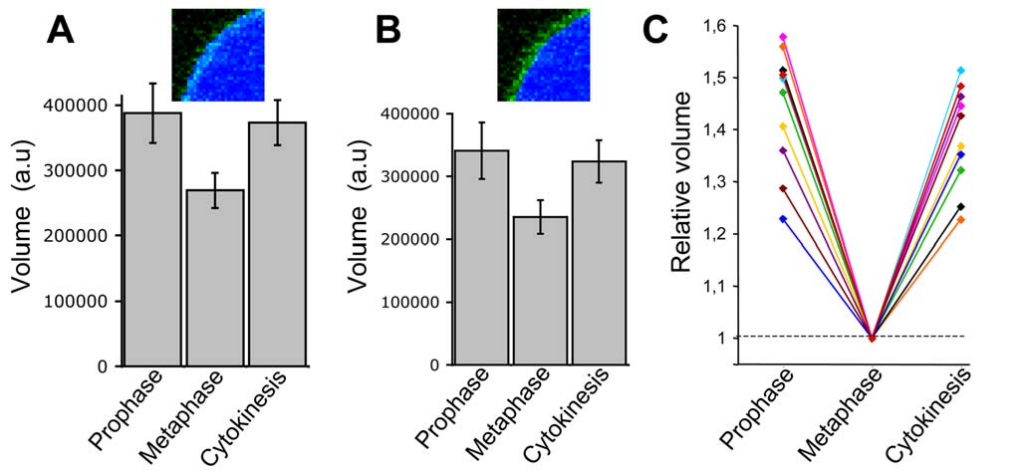
Changes of cell volume during mitosis. (A) Population analysis (average +/− standard deviation) of cell volume determined in 6 experiments for 10 single cells imaged during prophase, metaphase and last stages of cytokinesis. The most external pixels of the EGFP-CAAX signal were used to generate the mask (inset). The statistical significance of the volume difference between the cell cycle stages was calculated using the t-Student test (p<0.001). (B) Same analysis than in (A), but with the most internal pixels of the EGFP-CAAX signal were used to generate the mask (inset). (C) Individual representation of the volume data presented in (A). To facilitate the comparisons, the volume of each cell during metaphase was normalized to a relative value of 1.

The volumes thus obtained from 10 different cells imaged during 6 independent experiments were followed as the cells divide. Each of the cells showed a ∼20–50% volume loss (with an average of ∼30%, p<0.001) during the transition from prophase to metaphase, followed by full volume recovery towards the end of cytokinesis (Figure 2A). A possible source of error in the estimate of absolute volume derives from the mask assignment. For example, if we use the most internal pixels of the EGFP-CAAX signal to generate the mask (Figure 2B) it would result in a decrease of volume estimate of 12.1 ± 0.9 %, 12.1 ± 1.0 % and 13.1 ± 1.2 % during prophase, metaphase and cytokinesis, respectively. This potential imprecision has a minimal effect on the relative changes in volume (Figure 2B).

To facilitate the comparison between cells, we present the cell volume data normalized to its volume during metaphase, the stage at which the cell shape (sphere) and size (minimal) is the least variable (Figure 2C). The cell-to-cell variability probably reflects the uncertainty of timing at which the volume determination is performed during the transition from interphase to prometaphase, or from telophase to late stages of cytokinesis.

The volume decrease between prophase and metaphase is consistent with a recent study [3], also based on live cell imaging, where it was proposed that a “pre-mitotic condensation” process marks a clear reduction in cell volume before entering into mitosis. It was reported that this gradual condensation started during interphase, approximately 5 hrs before the execution of mitosis. Our observations complement theirs by showing that volume reduction occurs as cells undergo the prophase to metaphase transition, when their volume (this work and [3]) and surface area [1] are minimal.

One traditional approach to attain mean volume estimates within a population of cells combines light scattering with flow cytometry, where states along the cell cycle are defined by the DNA content of each cell. It has been shown that the volume of cells in G2+M (when the DNA content has doubled) is twice that of cells in G1 [4]. DNA content, however, is not sufficient to resolve cells in prophase from those in metaphase or cytokinesis (M). Thus, any volume determination carried out with this cell population will be inaccurate since it is strongly biased by the relative abundance of cells in G2, prophase and cytokinesis.

Cell mass (and hence volume) doubles before division. It has therefore been assumed that volume recovery occurs after the two daughter cells complete abscission and separate from each other. Unexpectedly we find a significant recovery of intracellular volume well before cell separation. Presumably the volume alterations reflect modulations in the isosmotic flow of water linked to ion transport, perhaps through channels such as those linked to the control of cell proliferation [5]; [6]; [7]. It remains to be established whether these changes in intracellular volume also occur in cells growing in tissues, and if so, what triggers them, what mechanisms are responsible for their regulation, and what, if any, is the importance to the physiology of the cell.

## Materials and Methods

### Cell synchronization and identification of cell stage

BSC1 monkey kidney epithelial cells stably expressing EGFP-CAAX [1] were grown as adherent cells in the presence of 0.5 mg/ml G418 in DMEM supplemented with 10% fetal bovine serum. The frequency of cells undergoing mitosis was increased to ∼20% by first keeping them for one day at 100% confluency, a condition that arrests them at the end of G1 [8]. Cells were then trypsinized and seeded onto #1.5 glass coverslips (25 mm in diameter) to approximately 50–70% confluency as a way to increase the fraction of cells entering S phase and imaged 18–20 h after [1].

The stage along the cell cycle was determined in the population of synchronous cells using phase contrast bright field illumination according to the following criteria: cells in prophase contain condensed chromosomes surrounded by the nuclear envelope; cells in metaphase appear round, lack their nuclear envelope and display condensed chromosomes aligned at the metaphase plate; cells undergoing cytokinesis (but before abscission) display a deep furrow, still have condensed chromosomes and start to show their nuclear envelope.

### Image acquisition

Cells were first washed with DMEM supplemented with 10% fetal bovine serum (FBS), followed by transfer of the glass coverslip to a sample holder (20/20 Technology, Inc.; Wilmington, NC) kept at 37 °C with 5% CO_2_ and 100% humidity) located inside an environmental chamber set at 37°C also containing the objective lenses. Imaging was done using α-MEM without phenol red supplemented with 20 mM HEPES, pH 7.2 and 5% FBS.

3D image imaging was done using a spinning disk confocal head (Perkin Elmer Co., Boston, MA) coupled to a fully motorized inverted microscope (Axiovert 200M, Carl Zeiss, Inc., Thornwood, NY) equipped with a 63 X lens (Pan Apochromat, 1.4 NA, Carl Zeiss, Inc). A 50 mW solid-state laser (473 nm; Crystal Laser, Reno, NV) coupled to the spinning head through an acoustic-optical tunable filter (AOTF) was used as light source. The imaging system operates under control of SlideBook 4.2 (Intelligent Imaging Innovations Inc, Denver, CO) and includes a computer controlled spherical aberration correction device (SAC, Intelligent Imaging Innovations, Inc, Denvers, CO) installed between the objective lens and the back illuminated CCD camera (Cascade 512B; Roper Scientific, Photometrics, Tuscon, AZ). Rapid acquisition of sequential optical sections spaced 0.25 µm apart was achieved with the aid of a piezo-driven stage (Applied Scientific Instrumentation, Eugene, OR). To reduce photo-bleaching the illumination was turned off during the readout period from the CCD to the computer.

### Volume calculation

Cell volume was estimated using SlideBook 4.2 by integrating trough out the cell height the area delimited in each imaging plane by the cellular outline, as defined by the location of EGFP-CAAX on the plasma membrane. Delineation of the cell boundary on each plane was achieved by applying two sequential filtering steps, a two-dimensional, nearest-neighbor deconvolution step to increase the signal-to-noise ratio of the boundary while at the same time evening out the local fluctuations in fluorescence signal, followed by a smoothening Gaussian 2-dimensional radial transformation (radius = 1 pixel). The sharp boundary thus generated defines the enclosed area on each plane, and its integration along the three-dimensional stack corresponds to the cell volume.
